# PRMT6 increases cytoplasmic localization of p21^CDKN1A^ in cancer cells through arginine methylation and makes more resistant to cytotoxic agents

**DOI:** 10.18632/oncotarget.5143

**Published:** 2015-09-03

**Authors:** Makoto Nakakido, Zhenzhong Deng, Takehiro Suzuki, Naoshi Dohmae, Yusuke Nakamura, Ryuji Hamamoto

**Affiliations:** ^1^ Section of Hematology/Oncology, Department of Medicine, The University of Chicago, MC2115, Chicago, IL 60637, USA; ^2^ Biomolecular Characterization Unit, RIKEN Center for Sustainable Resource Science, Saitama 351–0198, Japan

**Keywords:** p21^CDKN1A^, arginine methylation, PRMT6, subcellular localization, cancer chemoresistance

## Abstract

p21^CDKN1A^ is known as a potent inhibitor of cyclin-dependent kinase (CDK), which regulates cell cycle in response to various stimuli, including DNA damage, on the p53-dependent manner. Here we demonstrate that protein arginine methyltransferase 6 (PRMT6) methylates p21 at arginine 156 and promotes phosphorylation of threonine 145 on p21, resulting in the increase of cytoplasmic localization of p21. The cytoplasmic presence of p21 makes cancer cells more resistant to cytotoxic agents. Our results indicate that PRMT6 appears to be one of the key proteins to dysregulate p21 functions in human cancer, and targeting this pathway may be an appropriate strategy for development of anticancer drugs.

## INTRODUCTION

The cell cycle progression is strictly regulated through orchestrated functions by CDKs and CDK inhibitors [1, 2]. Cells possess several cell-cycle checkpoints to induce cell cycle arrest or to induce apoptosis when the genomic integrity is threatened, preventing the transmission of dangerous genetic mutations into subsequent cell generations [3]. p21^CDKN1A^ (WAF1/CIP1) was identified as a potent inhibitor of CDK [4], and is a key molecule to mediate p53-dependent G1 arrest [5]. In addition, by binding to Proliferating Cell Nuclear Antigen (PCNA), p21 interferes with PCNA-dependent DNA polymerase activity, thereby inhibiting DNA replication and also modulating various PCNA-dependent DNA repair processes [2, 6]. Accumulated evidence reveals that nuclear localization of p21 is necessary for these tumor suppressive functions [7]. Meanwhile, cytoplasmic p21 protein has been indicated to show an anti-apoptotic effect [7]. Cytoplasmic p21 protein is able to bind to and inhibit caspase 3, as well as the apoptotic kinases ASK1 and JNK [7–9]. These results imply that p21 protein may have distinct biological functions depending on its localization in the cell.

Both amount and activity of p21 protein are controlled by post-translational modifications. Three E3 ubiquitin ligase complexes, SCF^SKP2^ (SKP1-CUL1-SKP2), CRL4^CDT2^ (CUL4A or CUL4B-DDB1-CDT2) and APC/C^CDC20^ poly-ubiquitinate p21, and promote the proteasome-dependent proteolysis at specific cell cycle stages [2]. Tip 60-mediated acetylation is shown to counteract ubiquitination and stabilizes p21 protein [10]. In addition, multiple kinases have been reported to phosphorylate p21 and regulate its stability, protein-protein interactions and subcellular localization [11]. For example, Cyclin E/CDK2-mediated phosphorylation of p21 at serine 130 provides a docking site for SCF^SKP2^ E3 ubiquitin ligase complex, leading to proteasomal degradation [12]. Moreover, phosphorylation of p21 at serine 145 was reported to promote cytoplasmic localization of p21 [11, 13]. However, methylation of p21 has not been reported so far.

In the present study, we demonstrate that the protein arginine methyltransferase PRMT6, which is involved in human tumorigenesis [14, 15], methylates p21 at arginine 156 and promotes cytoplasmic localization of p21. In addition, we show evidence that translocation of p21 regulated by PRMT6-mediated arginine methylation affect chemosensitivity of cancer cells.

## RESULTS

### *In vitro* methylation of p21 by PRMT6

**Figure 1:**
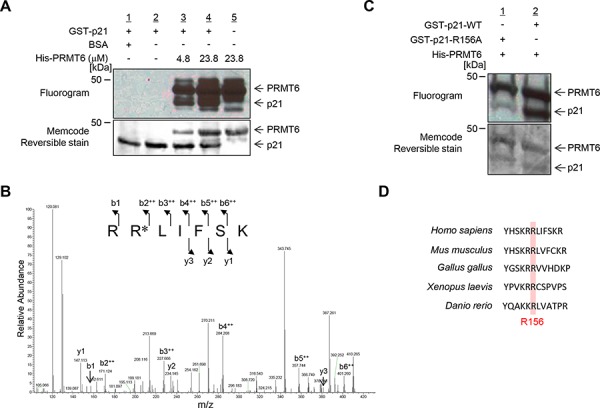
PRMT6 methylates p21 at arginine 156 *in vitro* **A.** *In vitro* methyltransferase assay. Recombinant p21 protein was methylated by PRMT6 in a dose-dependent manner. Methylated p21 was detected by fluorography and total proteins were stained with MemCode Reversible protein stain. **B.** LC-MS/MS spectrum of the dimethylated p21 (155–161) peptide. p21 recombinant protein was incubated with PRMT6 and SAM followed by separation by SDS-PAGE. An excised p21 band from the gel was digested with trypsin and subjected to LC-MS/MS analysis. The methylation site was determined by MASCOT search. **C.** Validation of the methylation on p21. Wild-type p21 (p21-WT) and mutant-type p21 (p21-R156A) were prepared using bacterial expression system and used for *in vitro* methyltransferase assay. **D.** Amino acid sequence alignment. Arginine 156 is conserved across these species.

In order to elucidate whether p21 serves as a substrate of protein methyltransferase reaction, we conducted *in vitro* methyltransferase assays using a variety of lysine and arginine methyltransferases that are shown to be involved in human tumorigenesis, and found that the arginine methyltransferase PRMT6 methylates p21 (Figure 1A and Supplementary Figure S1). Next, we examined liquid chromatography-tandem mass spectrometry (LC-MS/MS) analysis to identify an arginine residue(s) to be methylated by PRMT6 and found that arginine 156 on p21 was dimethylated by PRMT6 (Figure 1B and Supplementary Figure S2). To further verify the methylation, we prepared recombinant wild-type (WT) and arginine 156-substituted p21 (R156A) proteins, and performed an *in vitro* methyltransferase assay. The methylation signal was significantly diminished in p21-R156A protein compared to p21-WT protein (Figure 1C), indicating that p21 is methylated by PRMT6 at arginine 156 *in vitro*. This arginine residue is conserved among various species, implying the functional importance of this amino acid (Figure 1D).

### *In vivo* p21 methylation

**Figure 2:**
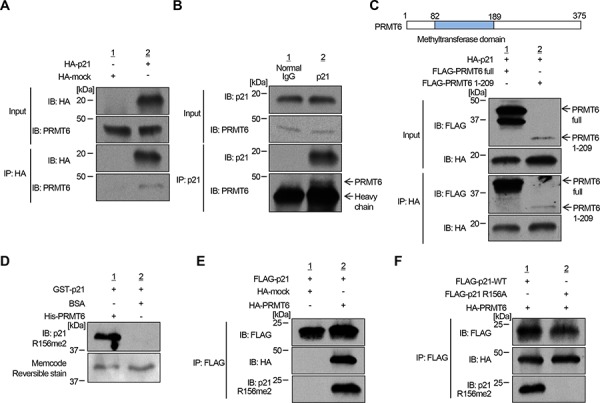
PRMT6 methylates p21 *in vivo* **A.** Interaction between exogenous HA-p21 and endogenous PRMT6 was confirmed by co-immunoprecipitation analysis. **B.** Confirmation of interactions between endogenous p21 and endogenous PRMT6 by co-immunoprecipitation analysis. **C.** N-terminal half fragment of PRMT6 binds to p21. Interaction of N-terminal half fragment of PRMT6 and exogenous p21 and was confirmed by co-immunoprecipitation analysis. **D.** Validation of anti-p21 R156me2 antibody. Recombinant p21 protein reacted with SAM and PRMT6 or BSA was analyzed by western blot analysis. Protein amount was visualized by MemCode Reversible staining. **E.** Confirmation of arginine 156 dimethylation in cells. 293T cells were co-transfected with FLAG-p21 and HA-PRMT6 or HA-mock vectors. Samples were prepared by immunoprecipitation using anti-FLAG M2 antibody-immobilized agarose beads and then analyzed by western blot analysis. **F.** FLAG-tagged wild-type or arginine 156-substituted p21 (R156A) was overexpressed in 293T cells. Immunoprecipitated samples were immunoblotted with anti-FLAG, anti-HA and anti-p21R156me2 antibodies.

**Figure 3:**
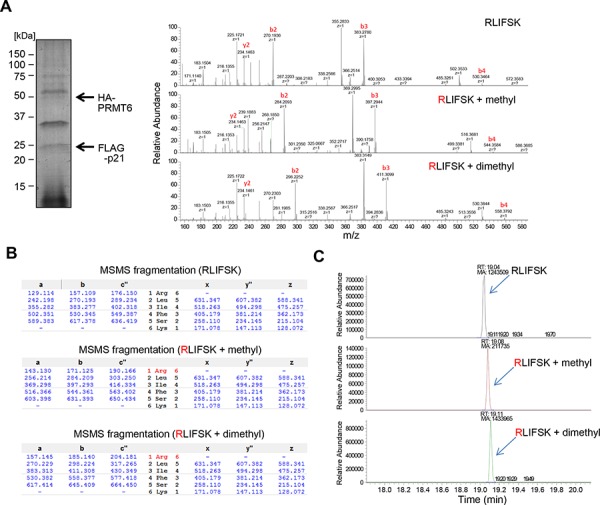
*In vivo* methylation of p21 was confirmed by LC-MS/MS analysis **A.** Wild-type p21 and PRMT6 were co-overexpressed and purified by immunoprecipitation followed by SDS-PAGE. Separated p21 protein was excised and digested in-gel with trypsin, and generated peptides were analyzed by LC-MS/MS. The MS-MS spectrum corresponding to the unmodified, monomethylated or dimethylated p21 156–161 peptide is shown. **B.** Theoretical values of both monomethylated and dimethylated fragments are summarized. **C.** Target MS/MS chromatograms show the presence of monomethylation as well as dimethylation.

We subsequently performed co-immunoprecipitation assays and confirmed the interaction between p21 and PRMT6 in both endogenous and exogenous levels (Figure 2A, 2B and Supplementary Figure S3). We also conducted co-immunoprecipitation analysis using a partial PRMT6 protein (aa 1–209) and found that p21 bound to the N-terminal half fragment of PRMT6 including the methyltransferase domain (Figure 2C). Then, to verify the methylation of p21 *in vivo*, we generated a polyclonal antibody that specifically recognizes dimethylated arginine 156 on p21 (p21 R156me2). Specificity of the antibody was confirmed by *in vitro* methyltransferase assay using recombinant p21 protein followed by western blot (Figure 2D). The methylation specific signal was observed when p21 protein was incubated with PRMT6, but not when it was incubated with BSA (Figure 2D), indicating that the antibody specifically recognizes methylated p21. Using this methylation-specific antibody, we examined methylation status of p21 *in vivo*. Firstly, we co-transfected FLAG-p21 together with HA-mock vector or with HA-PRMT6 expression vector into 293T cells. p21 proteins were purified by immunoprecipitation and subjected to western blot analysis using the methylation-specific antibody. As shown in Figure 2E, the signal intensity was significantly increased by co-transfection with PRMT6, indicating that p21 is methylated by PRMT6 *in vivo*. Subsequently, we introduced wild-type p21 or R156A-mutant p21 with PRMT6 in 293T cells, followed by immunoprecipitation and western blot analysis using the methylation-specific antibody. Then we confirmed that the methylation signal was abolished in R156A-mutant p21 (Figure 2F). To further validate PRMT6-mediated p21 methylation *in vivo*, we introduced both p21 and PRMT6 together in 293T cells, and purified the protein by immunoprecipitation (Figure 3A). The band corresponding to the FLAG-p21 was digested in gel with trypsin, and then the digestion mixture was subjected to LC-MS/MS analysis. The mass spectra with retention time 19.04, 19.09 and 19.11 indicate both monomethylation and dimethylation of R156 on p21 *in vivo* (Figure 3A and 3B). The selected ion current chromatograms of non-, mono- and dimethylated p21 peptides showed that the dimethylation seems to be a major methylation form of R156 on p21 (Figure 3C). This is consistent with the previous studies which showed that PRMT6 catalyzes asymmetric dimethylation, and also produces monomethylation on an arginine residue [14]. Taken together, these results demonstrate that p21 is methylated at arginine 156 by PRMT6 *in vivo*.

### Effect of PRMT6-mediated methylation on subcellular localization of p21

**Figure 4:**
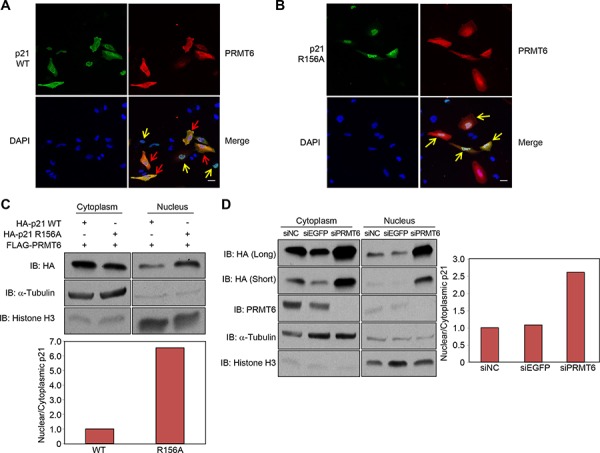
Subcellular localization of p21 was affected by the methylation **A, B.** Immunocytochemical analysis of HeLa cells transfected with wild-type or the arginine 156 substituted p21 and PRMT6 expression vectors. The cells expressing low levels of PRMT6 are highlighted by yellow arrows whereas the cells highly expressing PRMT6 are highlighted by red arrows. Scale bar, 30 μm. **C.** Wild-type or arginine 156 substituted p21 and PRMT6 were co-overexpressed in HCT116 p53^+/+^ cells. Nuclear and cytoplasmic fractions were prepared and analyzed by western blot. X-ray films were scanned by GS-800™ calibrated densitometer (Bio-Rad) and the signal intensity of p21 bands was analyzed by Quantity one software (Bio-Rad). The signal intensity of nuclear and cytoplasmic p21 was normalized by α-Tubulin and histone H3 signal, respectively. **D.** HCT116 p53^+/+^ cells were transfected with control siRNAs and PRMT6 siRNA. Nuclear and cytoplasmic fractions were prepared and analyzed by western blot. The signal intensity of nuclear and cytoplasmic p21 was normalized by α-Tubulin and histone H3 signal, respectively.

Since arginine methylation was suggested to regulate the nuclear/cytoplasmic shuttling of proteins [16], we co-introduced p21 and PRMT6 in HeLa cells, and examined subcellular localization of p21. Immunocytochemical analysis showed that p21 was mainly localized in the nucleus of the cells in which PRMT6 expression was absent or low, whereas cytoplasmic p21 was significantly increased in the cells in which PRMT6 was highly expressed (Figure 4A). On the contrary, R156A-mutant p21 was localized in the nucleus regardless to the PRMT6 expression (Figure 4B). In order to validate this result, we fractionated HCT116 cells overexpressing wild-type p21 or R156A-mutant p21, and quantified the amount of p21 protein in each fraction. We confirmed that the proportion of nuclear p21 was significantly high in R156A-ovrexpressing cells (Figure 4C), suggesting that the methylation at arginine 156 appears to enhance cytoplasmic localization of p21. To assess the effect of the methylation on p21 localization in the endogenous level, we examined the localization of p21 after PRMT6 knockdown. The proportion of the nuclear p21 protein was significantly elevated by siPRMT6 treatment, and interestingly, the total amount of p21 protein was remarkably increased (Figure 4D). Since *p21* mRNA levels were significantly increased after knockdown of PRMT6 (Supplementary Figure S4), it appears that PRMT6 represses p21 expression at the transcriptional level as concordant with a previous report [17]. These data indicate that PRMT6-mediated R156 methylation is likely to enhance cytoplasmic localization of p21.

### Molecular mechanism of p21 translocation promoted by PRMT6-mediated methylation

**Figure 5:**
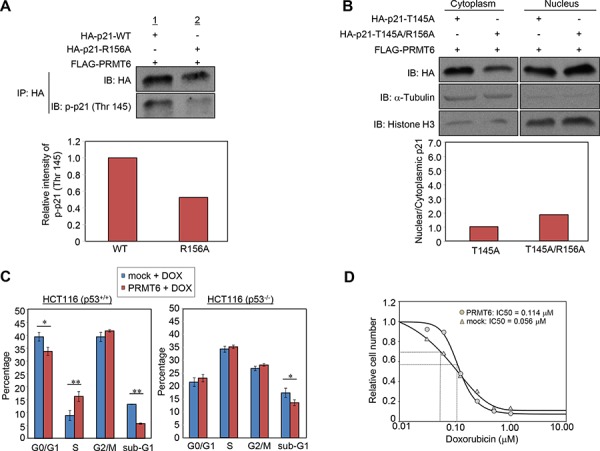
PRMT6-mediated p21 methylation affects p21 phosphorylation and chemosensitivity of cancer cells **A.** Wild-type or arginine 156-substituted p21 and PRMT6 were co-overexpressed in 293T cells and phosphorylation levels of the p21 proteins at threonine 145 were examined following immunoprecipitation. The signal intensity of phosphor-p21 (Thr 145) was quantified and normalized by p21 amount. **B.** Threonine 145-substituted p21 mutant (p21-T145A) or threonine 145/arginine 156 double substituted p21 mutant (p21-T145A/R156A) and PRMT6 were co-overexpressed in HCT 116 p53^+/+^ cells. Nuclear and cytoplasmic fractions were prepared and analyzed by western blot. The signal intensity of p21 bands in each fraction was quantified and normalized by that of α-Tubulin or histone H3. **C.** HCT 116 p53^+/+^ (left) or HCT116 p53^−/−^ (right) cells were transfected with mock or PRMT6 expression vector and incubated for 40 h. Subsequently, the cells were further incubated in the presence of 0.5 μM doxorubicin for additional 8 h. Cell cycle distribution was analyzed by flow cytometry coupled with BrdU staining. *P*-values were calculated with Student's *t* test (**P < 0.01, *P < 0.05). **D.** HCT116 p53^+/+^ cells transfected with a mock vector or a PRMT6 expression vector were treated with various concentrations of doxorubicin 24 h post transfection. Cell viability was measured 96 h after the drug treatment. IC50 was calculated using the SigmaPlot software.

It was reported that phosphorylation of p21 at threonine 145, which is located within the nuclear localization signal, regulates the p21 subcellular localization [11, 13]. Hence we hypothesized that the methylation at arginine 156 might affect the phosphorylation at threonine 145 and thereby influence the localization of p21. We then examined the phosphorylation levels of wild-type and R156A-mutant p21, and found that the phosphorylation was attenuated by R156A substitution (Figure 5A). To further validate the importance and interaction of the R156 methylation and T145 phosphorylation, we additionally prepared two mutant clones that contain T145A or T145A/R156A substitution, and examined subcellular localization. Consequently, we confirmed that the difference of nuclear/cytoplasmic portion between wild-type p21 and R156A mutant p21 became significantly small if threonine 145 was mutated (Figure 4C and 5B). These results imply that R156 methylation enhances phosphorylation of threonine 145 on p21, which appears to the mechanism to promote cytoplasmic localization of p21 in cancer cells.

### Effect of the PRMT6-mediated p21 methylation on anti-cancer drug sensitivity

Since p21 protein is a well-known cell-cycle regulator, we examined cell cycle regulation in response to DNA double strand breaks induced by doxorubicin, a strong inducer of p53 expression and p21 transactivation [18, 19], in the presence or absence of PRMT6 overexpression (Figure 5C, left). The proportion of cells at S phase increased while the cells at G0/G1 and sub-G1 phases were decreased by PRMT6 overexpression. Taken together with previous studies showing that nuclear p21 has a critical role to induce the G1 arrest and that cytoplasmic p21 causes chemoresistance of cancer cells by inhibiting proteins essential for apoptosis [20], this result indicates that the methylation-mediated p21 translocation appears to affect the regulation of cell cycle progression and apoptosis in response to DNA damage. Furthermore, the effect on cell cycle regulation induced by PRMT6 overexpression was not observed in p53 null cell lines (Figure 5C, right). Given p21 expression following DNA damage is induced by p53 [19], this result supports that the cell cycle regulation by PRMT6 overexpression is mediated by p21. We also evaluated the effect of PRMT6 overexpression on chemosensitivity and found that the IC50 of doxorubicin in PRMT6-overexpressing cells were significantly higher than that in control cells (Figure 5D). Taken together, p21 translocation promoted by PRMT6-mediated methylation appears to reduce chemosensitivity, which may cause chemoresistance of cancer cells.

## DISCUSSION

In this study, we demonstrated that PRMT6 methylates p21 at arginine 156 both *in vitro* and *in vivo,* and increases cytoplasmic localization of p21. Previous studies indicated that phosphorylation of threonine 145, which is located in the nuclear export signal motif, inhibits nuclear translocation of p21 [11, 13]. Our data imply that the PRMT6-mediated methylation then increase cytoplasmic localization of p21 through enhancement of the phosphorylation. Recently, Zhou *et al.* reported that cytoplasmic p21 induced by p65 prevents doxorubicin-mediated cell death in cancer cells [21], and accumulated knowledge clearly indicates that cytoplasmic p21 induces chemoresistant characteristics of cancer cells [22, 23]. Given that nuclear localization is required for cell-cycle arrest of cancer cells by p21, PRMT6-mediated R156 methylation may impair growth suppressive functions of p21 due to the promotion of its cytoplasmic subcellular localization in cancer cells.

PRMT6 has been considered as a histone arginine methyltransferase [24, 25] and reported to be predominantly localized in the nucleus [26, 27]. However, our results showed that PRMT6 is mainly localized in the cytoplasm in both exogenous and endogenous levels (Figure 4A, 4B and 4D), suggesting that the localization of PRMT6 may be altered based on the cell type and/or extrinsic stimuli. Even though whether PRMT6 methylates p21 in the nucleus or in the cytoplasm is still unknown, the present results imply that PRMT6 may play an important role in the functional regulation of not only histone proteins but also non-histone proteins as other histone methyltransferases [28–34].

Importantly, PRMT6 also suppresses p21 expression at the transcriptional level (Supplementary Figure S4) [17, 35]. Taken together with our findings, we suggest that PRMT6 may robustly inhibit the growth suppressive functions of p21 by two independent mechanisms; one is the transcriptional repression and the other is the increase of cytoplasmic localization of p21 through enhancement of the phosphorylation at T145, which is known to inhibit the translocation of p21 from cytoplasm to nucleus [2]. These results demonstrate that PRMT6 is a key protein to induce p21 dysfunctions in human cancer. Given that the induction of p21-mediated growth suppressive functions is considered as a critical mechanism for several anticancer agents such as HDAC inhibitors [36, 37] and that PRMT6 is significantly overexpressed in various types of cancer [15], PRMT6 is likely to be a promising anti-cancer drug target. Indeed, dysregulation of arginine and lysine methyltransferases have frequently been observed in human cancer [38–43], and these enzymes are now considered as emergent targets for anti-cancer drug development [14, 39, 44]. DNA-damaging chemotherapy in combination with a PRMT6 inhibitor is expected to improve the clinical outcome of anti-cancer treatment.

## MATERIALS AND METHODS

### Antibodies

The following primary antibodies were used: anti-FLAG (rabbit, F7425; Sigma-Aldrich; dilution used in WB: 1:3000), anti-FLAG (mouse, M2; Sigma-Aldrich; dilution used in ICC: 1:2000), anti-HA (rabbit, Y-11; Santa Cruz Biotechnology; dilution used in WB: 1:1000, ICC: 1:1000), anti-p21 (rabbit, 12D1; Cell Signaling Technology; dilution used in WB: 1:1000), anti-p21 (mouse, DCS60; Cell Signaling Technology; dilution used in immune-precipitation: 1:50), anti-PRMT6 (rabbit, D5A2N; Cell Signaling Technology; dilution used in WB: 1:1000), anti-α-Tubulin (mouse, DM1A; CALBIOCHEM; dilution used in WB: 1:1000), anti-histone H3 (rabbit, ab1791; abcam; dilution used in WB: 1:10000), anti-phosphor p21 (Thr 145) (rabbit, PA5–12646; Pierce; dilution used in WB: 1:1000). An anti-R156 dimethylated p21 antibody (Thermo Fisher Scientific; dilution used in WB: 1:500) was produced in rabbit immunized with a synthetic peptide.

### Cell culture

HeLa and 293T cell lines were obtained from American Type Culture Collection (ATCC) in 2014, and detailed information of DNA profile is described in Supplementary Table S1. HCT116 p53^−/−^ and HCT116 p53^+/+^ cell lines were obtained from Dr. Bert Vogelstein (Johns Hopkins University, Baltimore, MD). All cell lines were grown in monolayers in appropriate media supplemented with 10% fetal bovine serum and 1% antibiobic/antimycotic solution (Sigma-Aldrich): Dulbecco's modified Eagle's medium (D-MEM) for 293T cells; Eagle's minimal essential medium (E-MEM) for HeLa cells; McCoy's 5A medium for HCT116 cells. All cells were maintained at 37°C in humid air with 5% CO_2_ condition. Cells were transfected with FuGENE6 or Fugene HD (Promega) according to manufacturer's protocols.

### *In vitro* methyltransferase assay

*In vitro* methyltransferase assays were described previously [29, 30]. Briefly, recombinant p21 protein was incubated with recombinant PRMT6 protein and 2 μCi S-adenosyl-L-[methyl-^3^H]-methionine (Perkin Elmer) in a mixture of methylase activity buffer (50 mM Tris-HCl at pH 8.8, 10 mM DTT and 10 mM MgCl_2_) for 1 h at 30°C. After denaturing, samples were separated by SDS-PAGE, blotted to PVDF membrane and visualized by MemCode Reversible Stain (Thermo Fisher Scientific) and fluorography.

### Mass spectrometry

The reaction mixture of *in vitro* methyltransferase assay was subjected to SDS-PAGE, and the bands on the gel were visualized by SimplyBlue™ SafeStain (Thermo Fisher Scientific). The bands corresponding to p21 were excised from the gel, and digested with sequencing grade TPCK-trypsin (Worthington Biochemical) in 30 μL of digestion buffer (10 mM Tris-HCl, 0.05% decyl glucoside, pH 8.0) at 37°C for 12 h. The digest mixture was separated using a nanoflow LC (Easy nLC, Thermo Fisher Scientific) on an NTCC analytical column (C18, Φ.075 × 100 mm, 3 μm, Nikkyo Technos) with a linear gradient of 35% buffer B (100% acetonitrile and 0.1% formic acid) at a flow rate of 300 nL/min over 10 min, and subjected on-line to a Q-Exactive mass spectrometer (Thermo Fisher Scientific) with a nanospray ion source using data dependent TOP10 method. The MS/MS spectra were searched against the in-house database using local MASCOT server (version 2.3; Matrix Sciences). The quantitative analysis using Qual Browser (version 2.2; Thermo Fisher Scientific) was performed as described previously [30].

### Expression vector construction

p21 and PRMT6 genes are amplified from total human DNA standard prepared by reverse transcription from qPCR Human Reference Total RNA (Clontech) using KOD DNA polymerase (TOYOBO) and cloned into pCAGGSn3FC or pCAGGSnHC vectors. Mutations were introduced using Q5 site direct mutagenesis kit (New England Biolabs) according to the manufacture's protocol.

### Immunoprecipitation

293T cells were lysed 48 hrs after transfection with CelLytic M cell lysis reagent (Sigma-Aldrich) containing a complete protease inhibitor cocktail (Roche Applied Science). For FLAG or HA tagged protein, whole-cell extract was incubated with anti-FLAG M2 antibody conjugated agarose beads (Sigma-Aldrich) or anti HA antibody conjugated agarose beads (Sigma-Aldrich) at 4°C overnight. Following three times washing with PBS, proteins bound to the beads were eluted by incubating with FLAG or HA peptide at 4°C for 1 h. For endogenous protein, whole-cell extract was incubated with a primary antibody at 4°C overnight. Protein A/G conjugated agarose beads were added to bind proteins / antibody complex followed by washing three times with PBS. Proteins bound to the beads were eluted by boiling in Lane Marker Reducing Sample Buffer (Thermo Fisher Scientific).

### Western blot

Cell lysate samples were prepared from the cells lysed with CelLytic M lysis reagent (Sigma-Aldrich) supplemented with complete protease inhibitor cocktail (Roche Applied Science). Whole cell lysates or immunoprecipitated samples were separated by SDS-PAGE and blotted to nitrocellurose membrane. Protein bands were detected by incubating with horseradish peroxidase (HRP)-conjugated antibodies (GE Healthcare) at room temperature for 1 h and visualizing with enhanced chemiluminescence (GE Healthcare).

### Cellular fractionation

Nuclear and cytoplasmic fractions were prepared from HCT116 p53^+/+^ cells 48 h after expression vectors or siRNAs transfection using NE-PER Nuclear and Cytoplasmic Extraction Reagents (Thermo Fisher Scientific) according to the manufacture's protocol.

### Small interfering RNA transfection

siRNA oligonucleotide duplexes were purchased from Sigma-Aldrich for targeting PRMT6 transcripts. siEGFP and siNegative control (siNC, Cosmo Bio), which is a mixture of three different oligonucleotide duplexes were used as control siRNAs. The siRNA sequences are summarized in Supplementary Table S2. siRNA duplexes were transfected with Lipofectamine RNAi max (Thermo Fisher Scientific).

### Quantitative real-time PCR

Specific primers for human *GAPDH* (housekeeping gene), *SDH* (housekeeping gene) and *p21* were designed (primer sequences in Supplementary Table S3). PCR reactions were performed using ViiA™ 7 real-time PCR system (Thermo Fisher Scientific) following the manufacture's protocol.

### Immunocytochemistry

Cells were fixed 48 hrs after transfection in 4% paraformaldehyde in PBS at 4°C for 1 h, permeabilized in 0.1% Triton X-100 (Sigma-Aldrich) for 3 min at room temperature and blocked with 3% BSA for 1 h at room temperature. Fixed cells were incubated with each of primary antibody overnight at 4°C followed by incubation with Alexa Fluor-conjugated secondary antibody (Thermo Fisher Scientific) [30, 45] and observed using Leica confocal microscopy (SP5 tandem Scanner Spectral 2-Photon Confocal).

### Cell cycle analysis by flow cytometry

Cell cycle analysis was conducted as previously described [46–50]. A 5′-bromo-2′-deoxyuridine (BrdU) flow kit (BD Biosciences) was used for sample preparation according to the manufacture's protocol. Briefly, cells were transfected with expression vectors for 48 h followed by incubation with BrdU for 40 min. Collected cells were fixed, permeabilized, treated with DNAse and stained with FITC conjugated anti-BrdU antibody and 7-Aminoactinomycin D. Cell cycle analysis was performed using BD™ LSR II (BD Biosciences) and FlowJo software.

### Chemosensitivity assay

HCT116 p53^+/+^ cells were transfected with a mock vector or a FLAG-PRMT6 expression vector using Fugene HD reagent (Promega). Cells were treated with various concentrations of doxorubicin 24 h after transfection and cultured for 96 h. Cell viability was measured using CCK-8 (Dojindo). IC50 was calculated using the SigmaPlot software.

## SUPPLEMENTARY FIGURES AND TABLES


