# Supplementary material for: PRMT6 increases cytoplasmic localization of p21CDKN1A in cancer cells through arginine methylation and makes more resistant to cytotoxic agents
Source: Oncotarget. 2015 Sep 3;6(31):30957–67. doi: 10.18632/oncotarget.5143 (PMC4741580; doi:10.18632/oncotarget.5143)
Supplement: Supplementary file 1 [file oncotarget-06-30957-s001.pdf]

SUPPLEMENTARY FIGURES AND TABLES

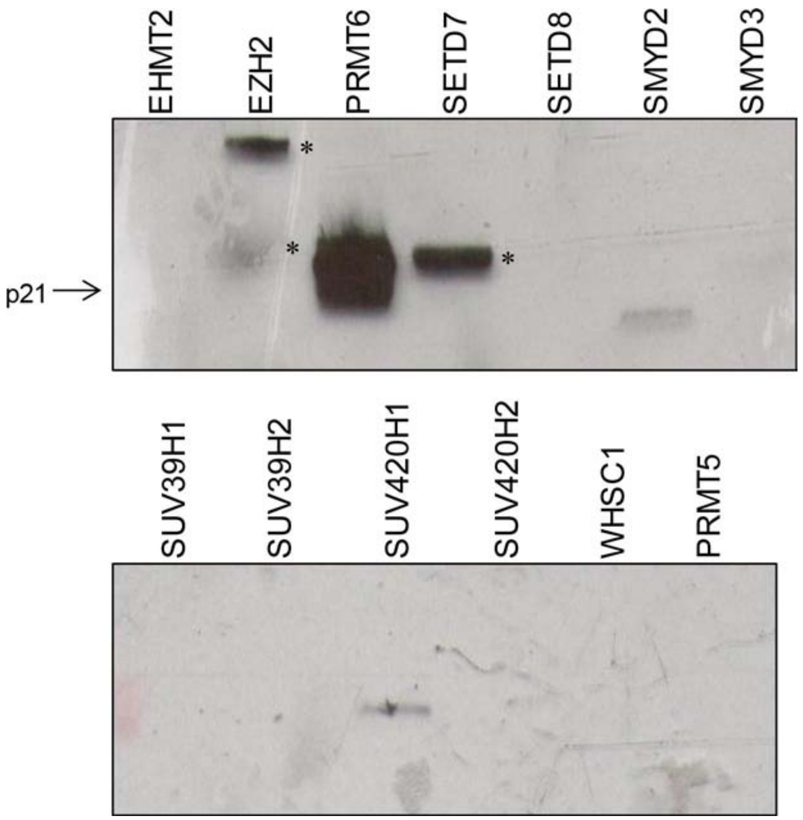

Supplementary Figure S1: PRMT6 methylates p21 *in vitro*. Recombinant p21 protein was incubated with a variety of methyltransferases, and methylaton signal was detected by autoradiography. *: Automethylation.

a	b	c"				x	y"	z
129.114	157.109	176.150	1	Arg	7	–	–	–
313.246	341.241	360.282	2	Arg	6	815.479	791.514	772.473
426.330	454.325	473.367	3	Leu	5	631.347	607.382	588.341
539.415	567.409	586.451	4	Ile	4	518.263	494.298	475.257
686.483	714.478	733.519	5	Phe	3	405.179	381.214	362.173
773.515	801.510	820.551	6	Ser	2	258.110	234.145	215.104
–	–	–	7	Lys	1	171.078	147.113	128.072
Ion charge state: 2								
65.061	79.058	88.579	1	Arg	7	–	–	–
157.127	171.124	180.645	2	Arg	6	408.243	396.261	386.740
213.669	227.666	237.187	3	Leu	5	316.177	304.195	294.674
270.211	284.208	293.729	4	Ile	4	259.635	247.653	238.132
343.745	357.743	367.263	5	Phe	3	203.093	191.111	181.590
387.261	401.259	410.779	6	Ser	2	129.559	117.576	108.056
–	–	–	7	Lys	1	86.043	74.060	64.540

Supplementary Figure S2: LC-MS/MS analysis showed methylation of p21 at arginine 156. Theoretical values of MS fragments are summarized.

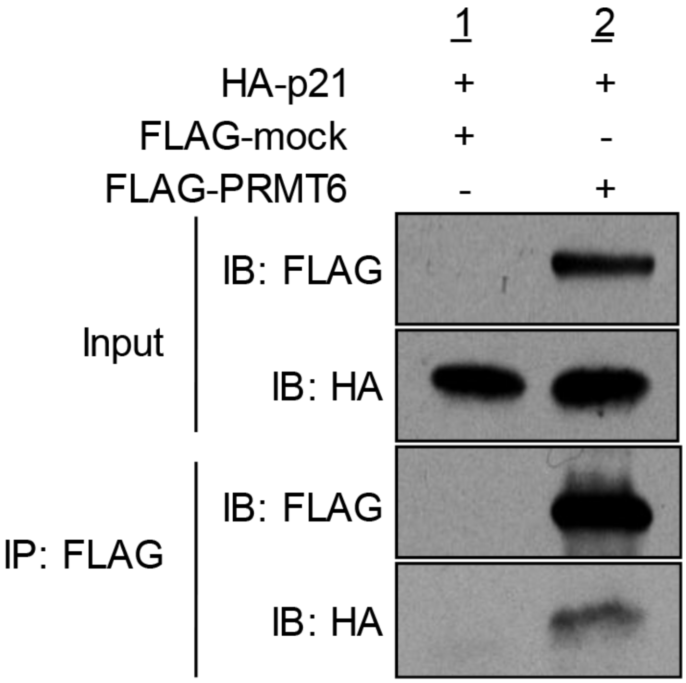

Supplementary Figure S3: PRMT6 binds to p21 in cells. The interaction of exogenous p21 and exogenous PRMT6 was confirmed by co-immunoprecipitation analysis.

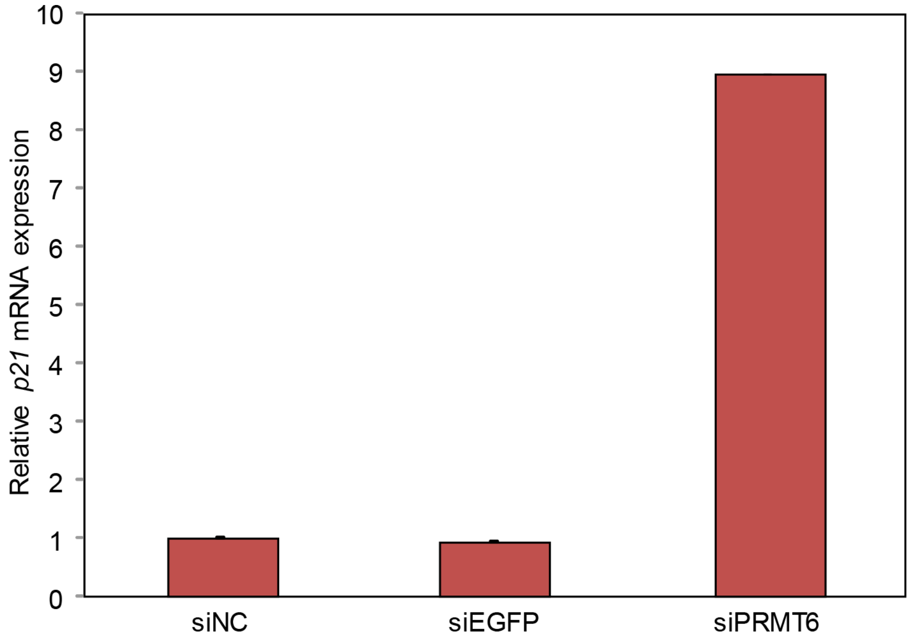

Supplementary Figure S4: p21 expression was transcriptionally increased by PRMT6 knockdown. Relative p21 mRNA levels were analyzed by qRT-PCR.

Supplementary Table S1. Information of certificated cell lines

Name	Certification institution	Tested method	DNA profile
**293T**	ATCC	STR	Amelogenin: X CSF1PO: 11, 12 D13S317: 12, 14 D16S539: 9, 13 D5S818: 8, 9 D7S820: 11 THO1: 7, 9.3 TPOX: 11 vWA: 16, 18, 19
**HeLa**	ATCC	STR	Amelogenin: X,Y CSF1PO: 11,12 D13S317: 11,14 D16S539: 9,11 D5S818: 11,12 D7S820: 10,11 THO1: 8 TPOX: 8 vWA: 15

ATCC; American Type Culture Collection

Supplementary Table S2. siRNA sequences

siRNA name		Sequence	
siEGFP		Sense	GCAGCACGACUUCUUCAAG
		Antisense	CUUGAAGAAGUCGUGCUGC
siNegative control (cocktail)	Target #1	Sense	AUCCGCGCGAUAGUACGUA
		Antisense	UACGUACUAUCGCGCGGAU
	Target #2	Sense	UUACGCGUAGCGUAAUACG
		Antisense	CGUAUUACGCUACGCGUAA
	Target #3	Sense	UAUUCGCGCGUAUAGCGGU
		Antisense	ACCGCUAUACGCGCGAAUA
siPRMT6		Sense	CCAUGCAUGGCUUUGCCAU
		Antisense	AUGGCAAAGCCAUGCAUGG

Supplementary Table S3. Primer sequences for quantitative RT-PCR

Gene name	Primer sequence
GAPDH (housekeeping gene)-f	5′ GCAAATTCCATGGCACCGTC 3′
GAPDH (housekeeping gene)-r	5′ TCGCCCCACTTGATTTTGG 3′
SDH (housekeeping gene)-f	5′ TGGGAACAAGAGGGCATCTG 3′
SDH (housekeeping gene)-r	5′ CCACCACTGCATCAAATTCATG 3′
p21–f	5′ GGAAGACCATGTGGACCTGT 3′
p21–r	5′ GGCGTTTGGAGTGGTAGAAA 3′
